# Genome and Transcriptome Sequencing Analysis of Fusarium *commune* Provides Insights into the Pathogenic Mechanisms of the Lotus Rhizome Rot

**DOI:** 10.1128/spectrum.00175-22

**Published:** 2022-07-05

**Authors:** Weigang Kuang, Lianhu Zhang, Lifang Ye, Jian Ma, Xugen Shi, Yachun Lin, Xiaotang Sun, Ruqiang Cui

**Affiliations:** a College of Agronomy, Jiangxi Agricultural University, Nanchang, Jiangxi, China; University of California Davis

**Keywords:** *Fusarium commune*, lotus, comparative genomics, transcriptome, plant-microbe interactions, effector

## Abstract

Fusarium wilt, a vascular wilt caused by *F. commune*, has been a serious problem for the lotus. Although some *F. commune* isolate genomes have been sequenced, little is known about the genomic information of the strain that causes Fusarium wilt of aquatic plants. In this study, the genome of *F. commune* FCN23 isolated from lotuses in China was sequenced using Illumina and PacBio sequencing platforms. The FCN23 genome consisted of 53 scaffolds with a combined size of 46,211,149 bp. According to the reference genome, F. oxysporum f. sp. *lycopersici* 4287 isolated from tomato, it was finally assembled into 14 putative chromosomes, including 10 core and 4 lineage-specific chromosomes. The genome contains about 3.45% repeats and encodes 14,698 putative protein-coding genes. Among these, 1,038 and 296 proteins were potentially secreted proteins and candidate effector proteins, respectively. Comparative genomic analysis showed that the CAZyme-coding genes and secondary metabolite biosynthesis genes of FCN23 were similar to those of other Ascomycetes. Additionally, the transcriptome of FCN23 during infection of lotus was analyzed and 7,013 differentially expressed genes were identified. Eight putative effectors that were upregulated in the infection stage were cloned. Among them, F23a002499 exhibited strong hypersensitive response after transiently expressed in Nicotiana benthamiana leaves. Our results provide a valuable genetic basis for understanding the molecular mechanism of the interaction between *F. commune* and aquatic plants.

**IMPORTANCE** Fusarium *commune* is an important soilborne pathogen with a wide range of hosts and can cause Fusarium wilt of land plants. However, there are few studies on Fusarium wilt of aquatic plants. Lotus rhizome rot mainly caused by *F. commune* is a devastating disease that causes extensive yield and quality losses in China. Here, we obtained high-quality genomic information of the FCN23 using Illumina NovaSeq and the third-generation sequencing technology PacBio Sequel II. Compared to the reference genome F. oxysporum f. sp. *lycopersici* strain 4287, it contains 11 core and 3 lineage-specific chromosomes. Many differentially expressed genes associated with pathogenicity were identified by RNA sequencing. The genome and transcriptome sequences of FCN23 will provide important genomic information and insights into the infection mechanisms of *F. commune* on aquatic plants.

## INTRODUCTION

Lotus (*Nelumbo nucifera* Gaertn.), belonging to Nelumbonaceae, is a perennial aquatic plant. It is an ornamental and edible plant in Asian countries, such as China, Japan, and India (1). It is a well-known and economically important plant, which cultivated for more than 2,000 years (2). The rhizome is used as a vegetable, and part of the leaves, flowers and fruits can be made into food and medicinal materials (3). Although the lotus is an aquatic plant, about 25 species of plant parasitic fungi have been recorded to have successfully colonized the rhizome and leaves of the lotus (4, 5). Other pathogenic microorganisms, such as *Hirschmanniella imamuri* and *H. diversa*, are known to cause serious damage to Indian lotus and are considered a major nematode threat to lotus production in Tokushima Prefecture, Japan (6).

Fusarium wilt is a common vascular fungal disease in plants, which can result in significant yield losses. Fusarium *commune* is one of the species associated with wilt and root rot. It is a recently characterized species closely related to the sister group F. oxysporum species complex (7). The *F. commune* in the soil initially invades the roots asymptomatically, and then colonizes the vascular tissues, causing wilting, necrosis, and yellowing of the above-ground parts. Cultivating a single crop for a long period on the same plot easily leads to the accumulation of pathogens and aggravates the occurrence of Fusarium wilt (8). Rhizome rot was first reported to be caused by F. oxysporum Schl. f. sp. *nelumbicola* (Nis. & Wat.) Booth in 1953 (9, 10). Recent studies show that the main pathogen was *F. commune* and has been a serious problem for lotuses in China (9, 11–13). The lotus will be damaged by Fusarium wilt throughout its growth period, causing the underground stems to rot and the ground to wither. Due to the consecutive monoculture of lotus plants in most parts of China, the disease has become more and more serious. Symptoms of wilt usually begin to appear in early June in southern China. When the disease is serious, the value of lotus products can be reduced by more than 60%, or even no harvest.

To date, the studies on Fusarium wilt have focused mainly on terrestrial plants, with a lack of investigation in aquatic plants. The mechanism of replant disease was still unknown in aquatic crops. Therefore, it is of great significance to investigate the interaction mechanism between the lotus and *F. commune* for the comprehensive prevention and control of the disease. However, the understanding of the mechanism is very limited as there are no relevant genetic data available for this pathogen. With the advantages of next-generation sequencing technology, the number of sequenced Fusarium genomes is increasing. The availablility of these sequences has made it possible to research *F. commune* at the genomic level. Genome sequencing of plant pathogens and comparison are effective methods to reveal the pathogenic mechanism and evolution of plant pathogens (14–16).

In this study, we sequenced the genome of *F. commune* used the PacBio and Illumina high-throughput sequencing technology, which is isolated from the aquatic lotus plant. Whole-genome and comparative genome analyses revealed that the genome encodes a diverse range of genes related to virulence, including carbohydrate-active enzymes, secreted proteins, effector proteins and genes involved in secondary metabolism. Our research has laid a solid foundation for exploring the pathogenic process of *F. commune* infecting aquatic plants.

## RESULTS

### Pathogen identification.

**FIG 1:**
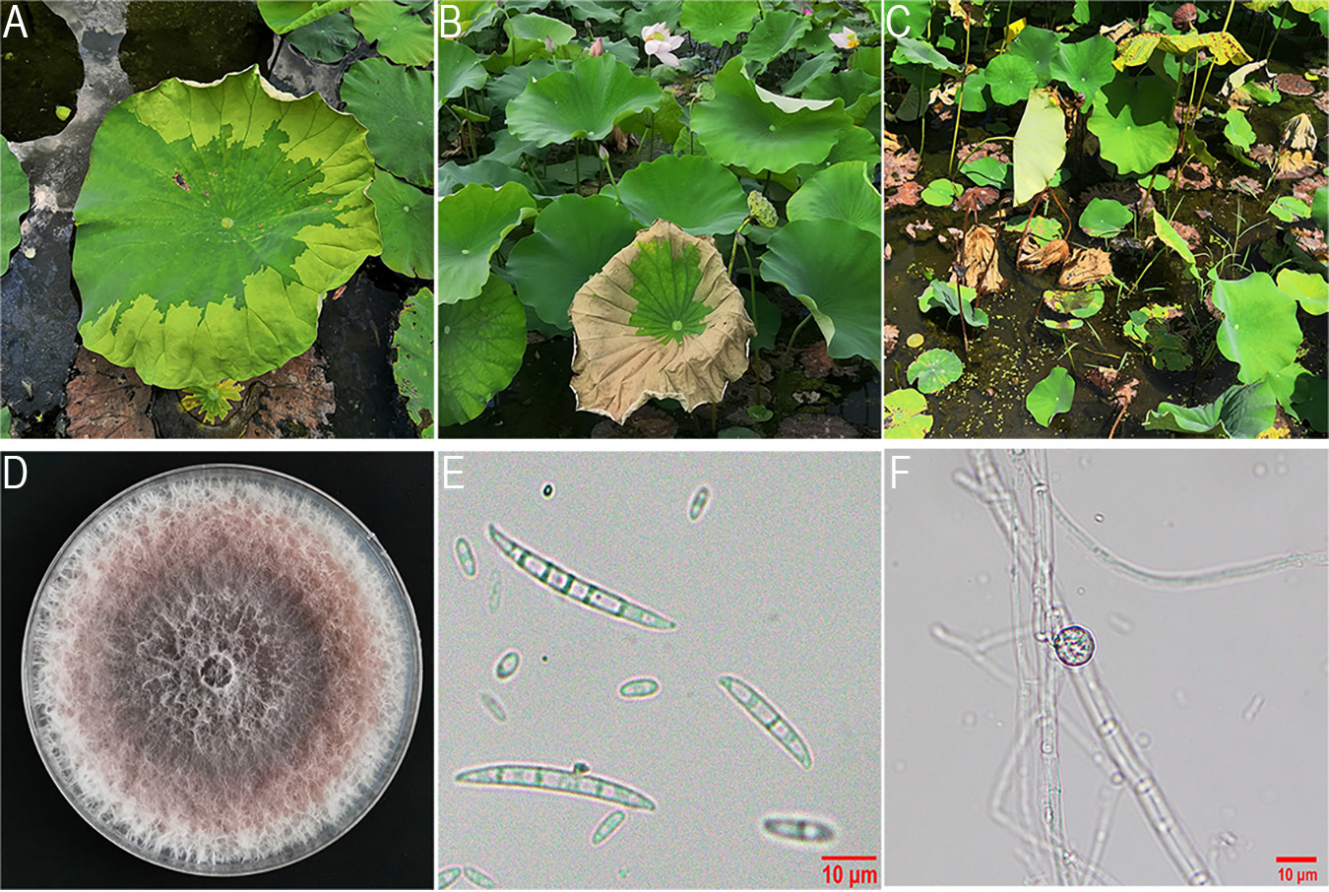
Symptoms of Fusarium wilt disease on lotus plants and morphological characteristics of strain FCN23. (A-C) Symptoms of Fusarium wilt disease on lotus plants in the early, middle, and late stages. (D) Mycelium colony on PDA medium at 25°C after 7 days of incubation. (E) Morphological characteristics of macroconidia and microconidium. (F) Morphological characteristics of chlamydospores.

Symptoms of Fusarium wilt disease on lotuses in the field and morphological characteristics of FCN23 strain are shown in Fig. 1. The colonies were characterized by an abundant white cottony mycelium, which became light to dark purple with age. Macroconidia had 3–5 septa, slightly curved at the apex, and measured 32.62 to 48.81 × 2.70 to 4.93 μm (*n* = 50). Microconidia had 0–1 septa, oval or ellipsoid, and were 5.33 to 8.38 × 2.05 to 3.64 μm (*n* = 50). Chlamydospores were spherical to oval. Phylogenic trees based on the sequences of the intergenic spacer region (IGS) and translation elongation factor 1-alpha (*EF-1α*) confirmed the isolate as *F. commune* (Fig. S1). In addition, a 295-bp size fragment was produced from FCN23 gDNA using specific PCR primers (efFc100F/efFc385R) for detecting *F. commune,* and no amplicon was detected using specific PCR primers (FOF1/FOR1) for F. oxysporum (Fig. S2). The typical symptoms appeared on all inoculated plants after 5 days of inoculation at 25°C, while the controls showed no symptoms (Fig. S3). The same pathogen was reisolated from the lesions, thereby fulfilling Koch’s postulates. These characteristics were consistent to those described for *F. commune*. Reisolations from infected plants confirmed that the reisolated pathogens possessed identical morphological characteristics to those of the original pathogens.

### Genome sequencing, assembly and annotation.

The genome of FCN23 was assembled from the data generated by the Illumina and PacBio sequencing platforms. After quality control of raw data, 9,570.6 Mb NovaSeq-data and 467,707,267 reads with an average length of 6,525 bp PacBio-data were generated. K-mer analysis confirmed the high quality of the library (Fig. S4). As showed in Table 1, the assembled FCN23 genome consisted of 53 scaffolds with an N50 length of 2,194,425 bp and a combined size of 46,211,149 bp. The average GC ratio and repeats rate were 47.55% and 3.45%, respectively. A total of 14,698 protein-coding genes were predicted with a total length of 21,370,401 bp. The average gene length was 1,454 bp, and the gene density was ~326 genes per one Mb of the genome. These predicted genes account for 46.25% of the genome. The genomic features for FCN23 are shown in Table 1.

**TABLE 1:** General features of the *F. commune* FCN23 genome

Features	FCN23
Size (bp)	46,211,149
Coverage (fold)	200x
%G+C content	47.55
Repeat rate (%)	3.45
N50 length (bp)	2,194,425
No. of scaffolds	53
Protein-coding genes	14,698
Gene total length (bp)	21,370,401
Avg gene length (bp)	1,454
Gene length/Genome (%)	46.25
Gene density (no. genes per Mb)	326

About 14,472 (98.46%), 3,976 (27.05%), 7,990 (54.36%), 4,781(32.53%), and 7,843 (53.36%) of the predicted genes presented homologies with known functions in the NCBI Non-Redundant (NR), Gene Ontology (GO), eggNOG, Kyoto Encyclopedia of Genes and Genomes (KEGG), and Swiss-Prot databases, respectively (Table S1 and S2). In addition, 303 tRNA and 88 rRNA were predicted in the assembly genome (Table S3). The repeat elements identified in FCN23 constituted the genome, including 2.9997% as interspersed repeats and 0.4554% as tandem repeats. The most abundant repetitive element was the DNA transposons (1.73%), followed by long terminal repeat (LTR) (0.93%), non-LTR retrotransposon LINEs (long interspersed nuclear elements) (0.27%), rolling circle (RC) (0.01%) (Table 2). Among the tandem repeats, a total 121,086 bp minisatellite was identified, accounting for 0.2620% of the genome. In addition, a 19,577 bp (0.0424%) microsatellite was identified. Compared with the reference genomes Fo47 (17) and Fol4287 (18), FCN23 has a smaller genome size and repeat rate, and fewer genes.

**TABLE 2:** General features of repeat element types in the FCN23 genome

Type	Total length (bp)	No. elements	Percentage in genome (%)
Long terminal repeat (LTR)	431,295	1,820	0.9333
DNA transposons	800,188	1,545	1.7316
Non-LTR retrotransposon (LINEs)	124,881	637	0.2702
Short interspersed repeated (SINE)	1,214	18	0.0026
Rolling circle (RC)	5,380	67	0.0116
Unknown	34,823	263	0.0754
Total interspersed repeated	1,386,185	4,350	2.9997
Minisatellite DNA	121,086	2,722	0.2620
Microsatellite DNA	19,577	476	0.0424
Total tandem repeats	210,429	3,688	0.4554

### Comparative genomic and orthologous clusters analysis.

**FIG 2:**
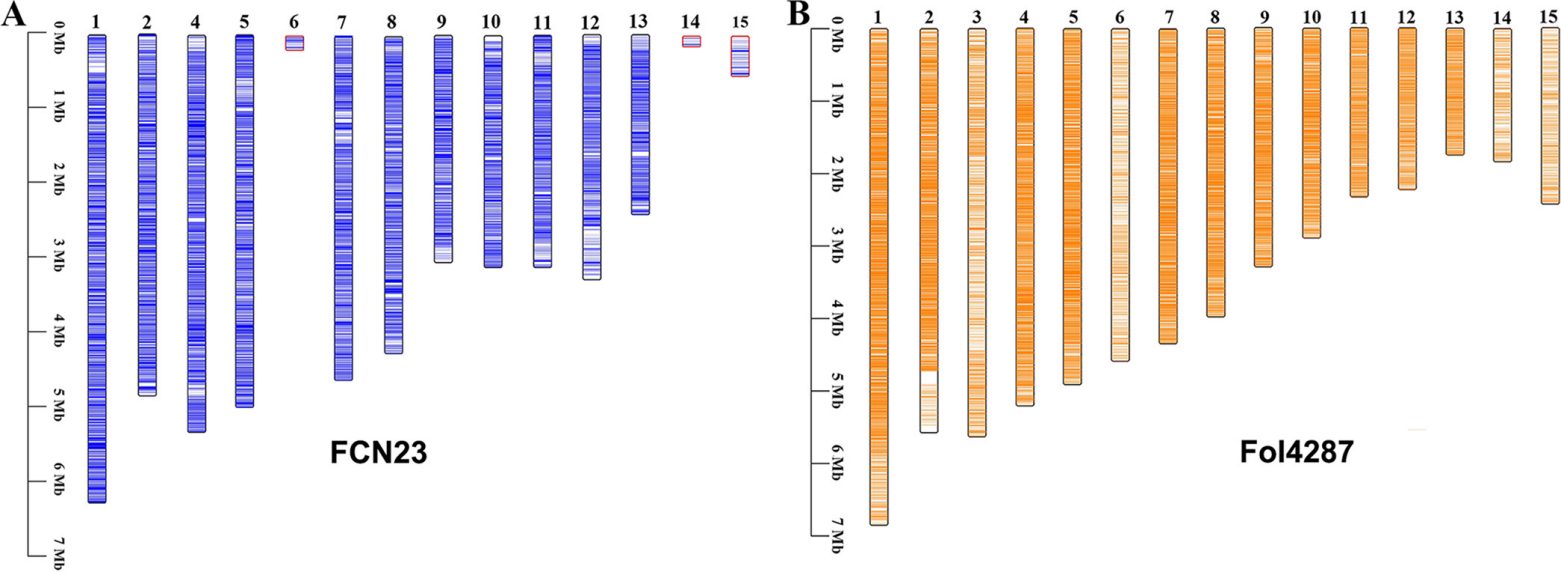
Comparison of gene distribution characteristics of core and lineage-specific chromosomes in FCN23 and Fol4287 genomes. (A) FCN23, Fusarium *commune* FCN23. (B) Fol4287, F. oxysporum f. sp. *lycopersici* 4287. Core chromosomes: 1, 2, 4, 5, 7, 8, 9, 10, 11, 12, and 13. Lineage-specific chromosomes: 3, 6, 14, and 15. The color represents the location of the gene.

Genomic sequences of FCN23 was aligned with reference genome sequences of the well characterized strain Fol4287 (GenBank accession number AAXH00000000.1). Based on the reference genome, putative core chromosomes 1, 2, 4, 5, 7, 8, 9, 10, 11, 12, and 13 were assembled. In addition, putative lineage-specific (LS) chromosomes 6, 14, and 15 were identified in the FCN23 genome. Lineage-specific genes present in FCN23 was distinct from those in Fol4287. The gene distribution characteristics of core and lineage-specific chromosomes in the FCN23 and Fol4287 genomes is presented in Fig. 2. We further verified the results of the putative chromosome assembly by PCR amplification. The results were consistent with the assembly results (Table S4 and Fig. S5).

**FIG 3:**
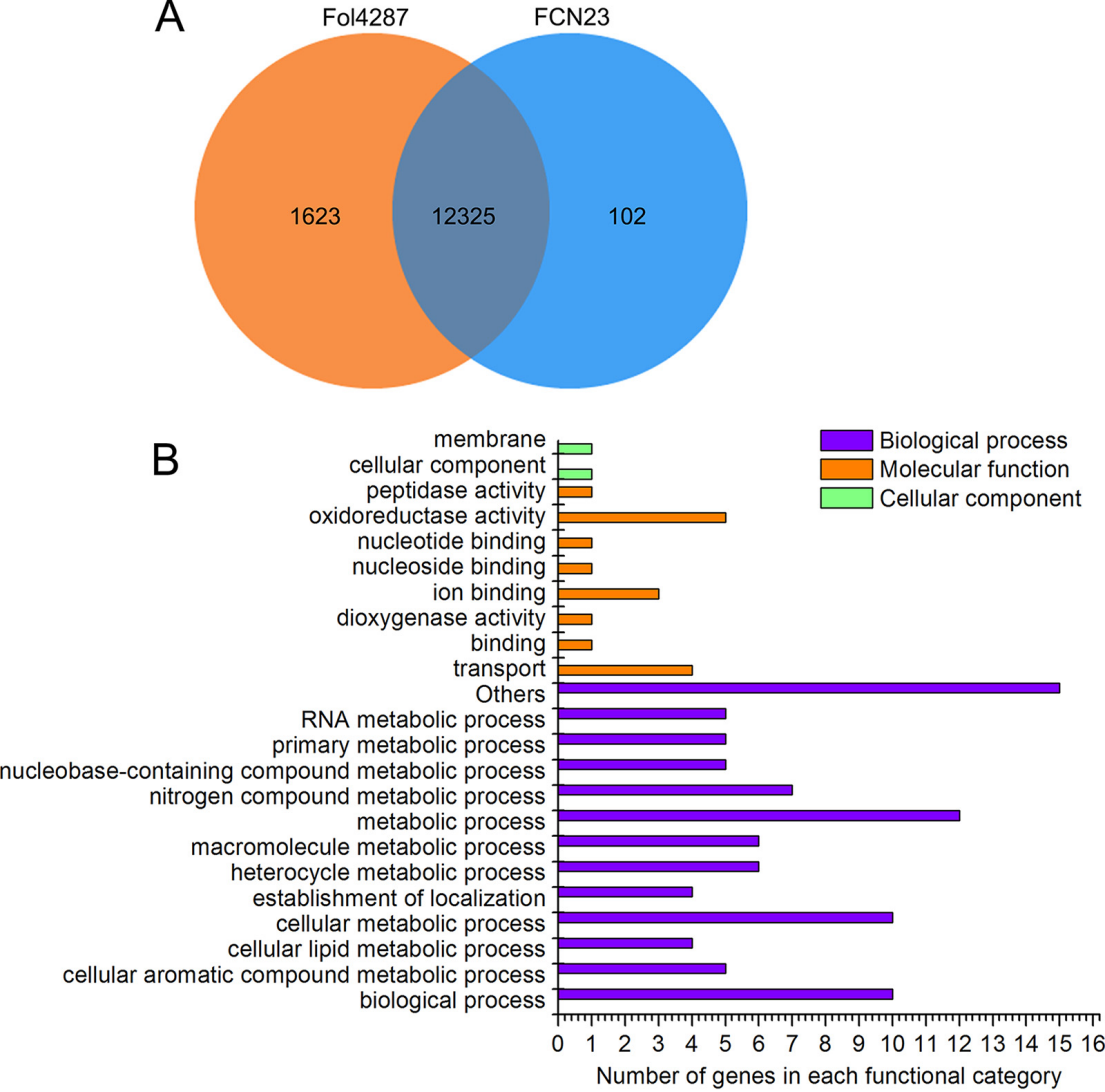
Comparison of orthologous genes, FCN23 and the reference F. oxysporum f. sp. *lycopersici* genome. (A) Venn diagram of gene clusters, FCN23, *F. commune*; Fol4287, F. oxysporum f. sp. *lycopersici* 4287. (B) GO enrichment analysis of annotated genes exists in FCN23 genome, but not in Fol4287.

The comparative analysis of FCN23 and Fol4287 showed that the two strains shared 12,325 orthologous clusters. In addition, 102 clusters containing 224 proteins were found to be unique to the FCN23 (Fig. 3A and Table S5). GO analysis among the group of 102 unique clusters showed an enrichment of genes associated with a nitrogen compound metabolic process, cellular metabolic process, and heterocycle metabolic process (Fig. 3B).

Similarly, we compared four genomes, including F. graminearum, *F. poae*, *F. verticillioides* and FCN23 using OrthoVenn. In total, 56,936 genes were grouped into 13,626 clusters. Among these clusters, 5,264 orthologous clusters (containing at least two species) and 8,362 single copy gene clusters were identified. A total of 8,814 core gene clusters were identified from the four species, as shown in Fig. S6. FCN23 contains 108 specific gene clusters, including 237 genes, of which 101 genes were enriched in the biological process and 15 genes were enriched in molecular function. These unique genes can be explored as a potential target to affect the metabolism and pathogenicity of FCN23 (Table S6). The genomic information of strains used for comparison in this study is shown in Table S7.

### Carbohydrate-active enzymes.

The structure and composition of plant cell walls are highly complex and diverse, mainly composed of cellulose, hemicellulose and pectin. Pathogenic fungi can decompose and utilize plant cell wall polysaccharides by carbohydrate-active enzymes. In total, 662 CAZyme-coding gene homologs were predicted by the domain-based annotation dbCAN in combination with CAT in the genome of FCN23, which comprised 325 glycoside hydrolases (GHs), 26 polysaccharide lyases (PLs), 54 carbohydrate esterases (CEs), 126 auxiliary activities (AAs), 107 glycosyl transferases (GTs), and 24 carbohydrate-binding modules (CBMs) (Table 3). Compared with other fungi, FCN23 has a larger potential glycoside hydrolase group, which may be an important virulence factor for its infection of the lotus (Table S8).

**TABLE 3:** Statistical summary of carbohydrate active enzymes in FCN23 and other 11 fungal genomes

CAZymes	AAs	CBMs	CEs	GHs	GTs	PLs	Total	Pathogen lifesyle
Ustilago maydis	88	6	49	265	113	5	526	Biotroph
*Puccinia graminis* f. sp. *tritici*	28	1	37	152	80	3	301	Biotroph
FCN23	126	24	54	325	107	26	662	Hemibiotroph
Fusarium graminearum	100	17	42	246	97	22	524	Hemibiotroph
Fusarium *poae*	100	17	44	238	106	19	524	Hemibiotroph
Fusarium *verticillioides*	112	14	49	290	97	23	585	Hemibiotroph
Magnaporthe oryzae	114	14	53	251	94	6	532	Hemibiotroph
Alternaria alternata	164	10	58	280	94	25	631	Hemibiotroph
Botrytis cinerea	109	20	41	274	107	10	561	Necrotroph
*Valsa mali*	104	10	32	266	88	13	513	Necrotroph
Verticillium dahliae	99	18	47	247	85	31	527	Necrotroph
Sclerotinia sclerotiorum	78	16	35	222	84	5	440	Necrotroph

### Prediction of secondary metabolite biosynthetic gene clusters.

**FIG 4:**
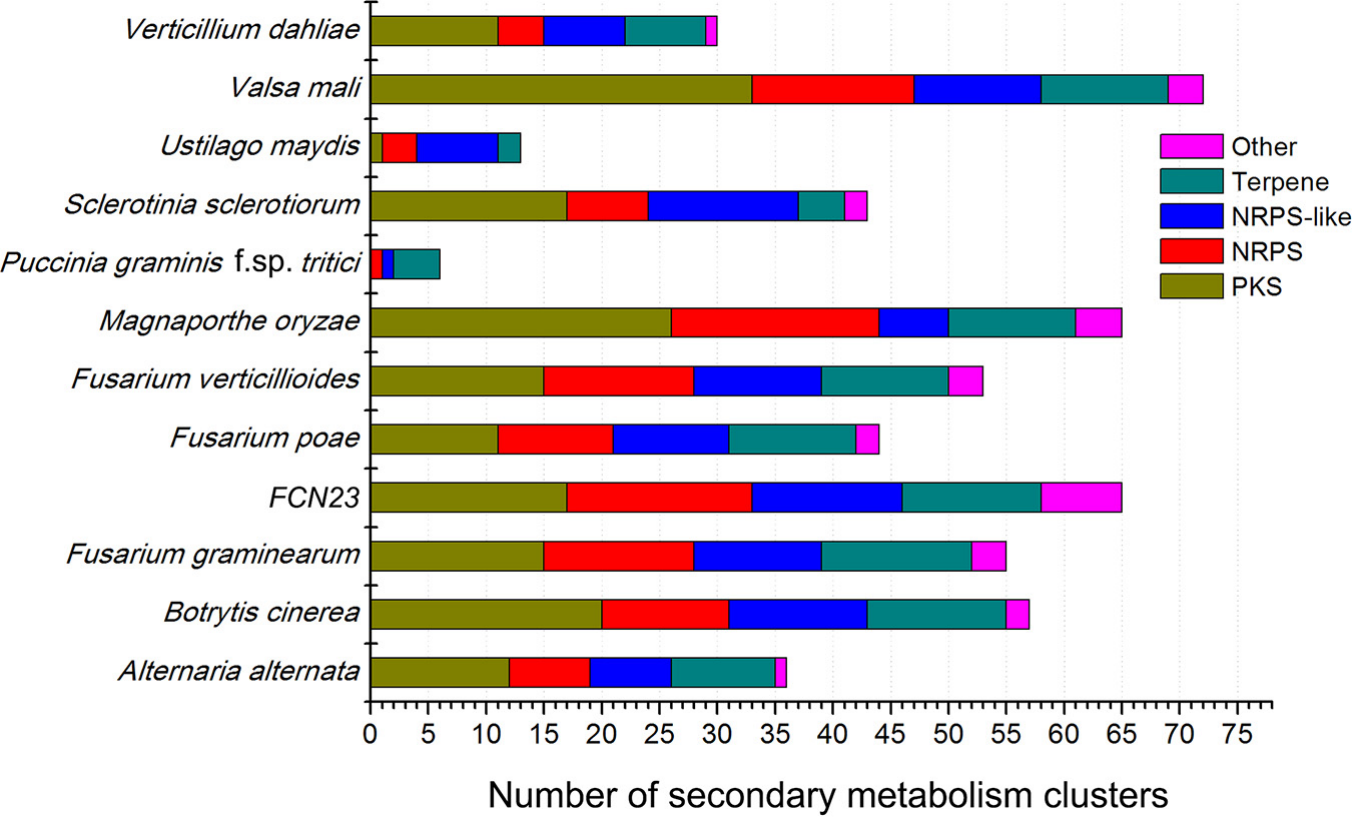
Comparison of secondary metabolism gene clusters, FCN23 and other fungi. PKS, polyketide synthase; NRPS, nonribosomal peptide synthetase; NRPS-like, NRPS-like fragment.

Fungi can produce many secondary metabolites, which have a variety of functions and great pharmacological potential (19). Related genes encoding enzymes that control the biosynthetic pathway of secondary metabolites are usually clustered and continuous in the fungal genome. In addition, the gene cluster for the synthesis of secondary metabolites usually also includes genes involved in the completion of the product metabolism pathway, transmembrane, and activation of related specific transcription factors. In the present study, a total of 65 gene clusters were distinguished in the strain FCN23 genome using antiSMASH software, including 1 tRNA-dependent cyclodipeptide synthases (CDPS), 2 betalactones, and 4 indoless. In addition, 29 nonribosomal peptide synthetases (NRPS), 17 polyketide synthases (PKS), and 12 terpene gene clusters were revealed, accounting for 89.23% of the total number of predicted gene clusters. Similar results were also observed in other ascomycetes and were more abundant than basidiomycetes. The details of the gene clusters in FCN23 and other fungi are displayed in Fig. 4.

### Comparative transcriptome analysis of host-pathogen interactions.

To analyze pathogenicity-associated genes of FCN23, RNA of infected lotus tissue and of pure mycelium were sequenced by paired-end sequencing on an Illumina NovaSeq 6000 platform. After trimming low-quality sequences, the cleaned RNA-seq reads were mapped to the reference genome of FCN23. The clean reads Q20 and Q30 value was higher than 93%. About 47.43 M and 43.70 M cleaned reads were obtained from the mycelium growth on medium and infected tissue, respectively. Approximately 97.37% and 38.43% of the clean reads were mapped to the FCN23 genome (Table S9). We performed correlation analysis between expression values of different samples. The Pearson’s correlation coeffcient of the three biological replicates in the experimental group (LotD) exceeded 0.91, and in the control group (FCN) exceeded 0.98; the Pearson’s correlation coeffcient between the experimental group and the control group was 0.40. The results showed higher similarities among samples with the same treatment (Fig. S7).

**FIG 5:**
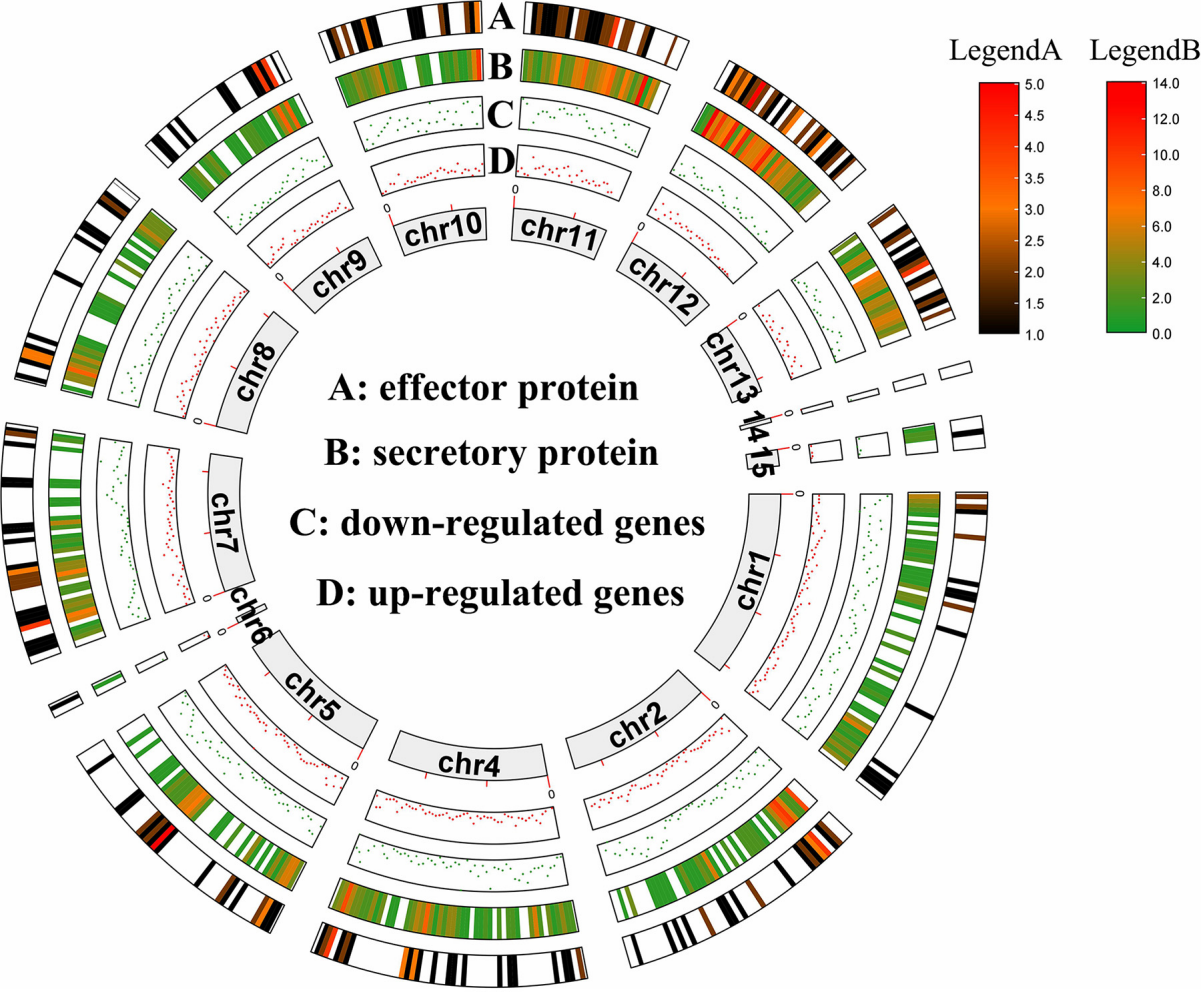
Global view of differentially-expressed genes, secreted proteins, and putative effector proteins in the FCN23 genome. (A) Chromosomal localization of putative effector proteins in the FCN23 genome. Color regions represent the location and number of putative effector proteins. (B) Chromosomal localization of putative secreted proteins. Color regions represent the location and number of putative secreted proteins. (C) The heat map of downregulated genes in FCN23 during infection of ‘Taikong lotus 36’. Each dot represents a gene. (D) The heat map of upregulated genes.

A total of 7,013 differentially expressed genes (DEGs) were identified, with 3,000 genes exhibiting a significant increase in abundance of transcripts during infection (Fig. S8). Through the analysis of DEGs locations, it is shown that the DEGs were mainly located on the core chromosome. Genomic mapping analysis showed that the DEGs were mainly located on the 11 core chromosomes (Fig. 5).

GO assignments were used for the functional classification of the upregulated genes. The three most basic ‘molecular function’ categories are transporter activity, transmembrane transporter activity, and coenzyme binding. The top three “biological processes” are the carbohydrate metabolic process, small molecule metabolic process, and organonitrogen compound biosynthetic process; and the top three “cell components” categories are cytoplasm, protein-containing complex, and cytoplasmic part.

**FIG 6:**
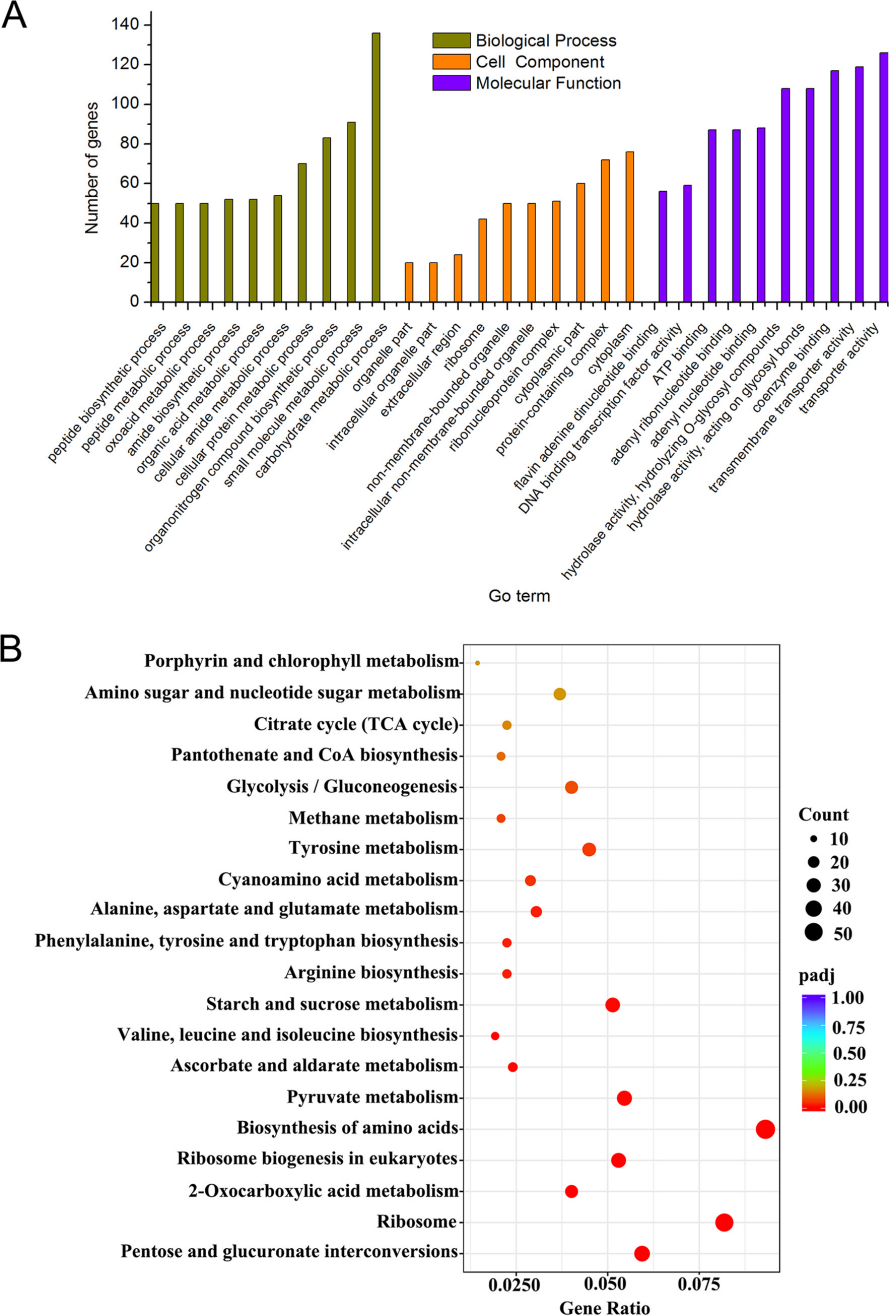
Enrichment analysis of upregulated differentially expressed genes. (A) Histogram of GO classifications. (B) KEGG enrichment scatter diagram.

To investigate biological pathways that are active during the fungal infection, the transcriptome of FCN23 was fit to the reference canonical pathways in KEGG. The 1,168 upregulated genes were mapped to 93 pathways. Among these pathways, ‘Biosynthesis of amino acids’ contained the highest percentage of genes (58 genes), followed by ‘Biosynthesis of cofactors’ and ‘Carbon metabolism’ (Fig. 6). These results suggest that FCN23 was active in secondary metabolite biosynthesis and catalytic activity during infection. Carbon metabolism plays an important role in cellulose degradation, and amino acid metabolism promotes humus synthesis.

### Prediction and functional analysis of secretory effector proteins.

In the present study, we first used SignalP to screen secretory proteins and identify 1,471 proteins with a secretion signal. Next, 1,218 proteins without transmembrane domains were identified in these proteins using TMHMM. Finally, 1,038 proteins contained glycophosphatidylinositol anchor motifs were predicted by PredGPI, accounting for 7.06% of FCN23 genome (Table S10). The region with the most amino acid length distribution was concentrated between 70 and 500 amino acid sequences, accounting for 69.56% of the putative secretory proteins.

We further analyzed these predicted secretory proteins and screened the possible effector proteins. Finally, 296 candidates of small-secreted effector proteins were predicted using EffectorP 3.0, accounting for 2.0% of the total proteins and 28.5% of the total secreted proteins (Table S11). Most of them were identified as a hypothetical protein or uncharacterized protein. Chromosomal localization of putative secreted and effector proteins are shown in Fig. 5.

**FIG 7:**
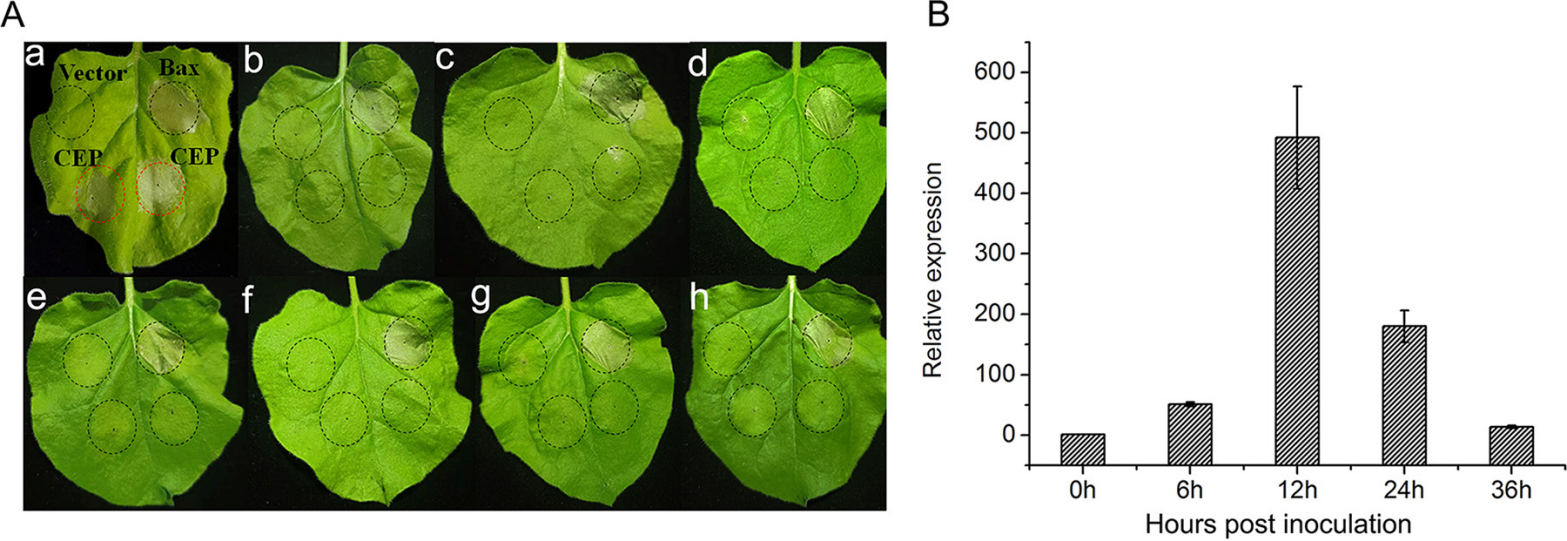
Functional analysis of putative FCN23 effectors. (A) Transient expression of the putative effector in N. benthamiana leaves via agro-infiltration. Leaves of N. benthamiana inoculated with *Agrobacteria* carrying putative effectors were assessed at 3 days postinoculation (positive control, Bax; negative control, pBinGFP); (a) F23a002499 induced hypersensitive reaction, (b-h) failed to induce hypersensitive reaction. (B) The dynamic expression patterns of the F23a002499 gene at 6, 12, 24, and 36 h postinoculation.

Fungal effectors play an important role in the interaction between pathogenic fungi and hosts, which directly affect the invasion, expansion, and disease occurrence of pathogenic fungi (20, 21). In addition, the function of most fungal effector proteins is unclear. In order to obtain information about the function of putative effectors, we selected eight candidate effector proteins (CEP) that were upregulated in the infection stage for transient expression in N. benthamiana. Primers used for functional analysis of putative effectors were listed in Table S12. F23a002499 shows a significant ability to trigger a hypersensitive response in plants, but all other seven candidate effectors do not, suggesting that F23a002499 is involved in the infection process of the pathogen (Fig. 7A). Quantitative RT-PCR analysis was applied to analyze the dynamic expression patterns of the F23a002499 gene at different time. The results showed that the expression of F23a002499 gene were highest at the early infection stage (especially at 12 h postinoculation) (Fig. 7B).

## DISCUSSION

Fusarium *commune* is a typical soilborne disease, which can cause Fusarium wilt of many plants. The lotus is an important aquatic crop of considerable agricultural, ornamental, religious, and medical importance in Asia. The rhizome rot of lotus plants caused by *F. commune* is one of the most common and destructive diseases of lotuses. However, the study of pathogenic mechanism is very limited. With the popularization of high-throughput sequencing technology, the genomes of more and more species have been sequenced, including many important plant pathogenic fungi. Genome sequencing has laid an important foundation for accelerating the study of pathogenic biology and pathogenesis. In this study, the genome of *F. commune* isolated from aquatic plant was assembled using short-read sequencing technologies combined with the PacBio long reads. The size of the genome was approximately 46,211,149 bp, which is smaller than the reference genome sequence isolate from other land crops (17, 18). The FCN23 genome contains 11 core and 3 LS chromosomes, that were distinct from Fol4287 F. oxysporum f. sp. *Lycopersici* and Fo47. The complete LS chromosome 3 was missing in FCN23 genome. Comparative genomic studies show that the genome of F. Oxysporum is composed of 10–11 core chromosomes and varying numbers of LS chromosomes (22). LS chromosomes are related to host-specific pathogenicity in phytopathogenic F. oxysporum (18). The differences of LS chromosomes 3, 6, 14, and 15 between the FCN23 and Fol4287 genomes may be caused by their host characteristics. These specific genomic differences can be explored as potential targets for studying the pathogenic mechanism of the FCN23.

The plant cell wall is the first barrier encountered by pathogens when they infect plants. The complexity of the plant cell wall also affected the diversity of CAZymes produced by pathogens. During the interaction between plant pathogenic fungi and their host, F. oxysporum will secrete a large number of CAZymes to overcome the plant cell wall, including pectinase, cellulase, xylanase, hemicellulose, and ligninase, etc. (23). CAZymes are conducive to the invasion, colonization and expansion of pathogenic fungi. There are 662 putative CAZyme-coding genes predicted in the FCN23 genome. The number is higher than that of the CAZymes in biotroph and necrotroph pathogens. The CAZyme profiles of FCN23 are close to that of soilborne pathogenic fungus, such as F. verticillioides and V. dahliae. Each of these CAZymes may play a role in different stages of the infection and expansion process. Although these enzymes may be important for the virulence of pathogens, they are a double-edged sword. When they are secreted into plants, they may cause plant defense reactions, such as callose deposition and programmed cell death (24, 25). Previous studies have shown that some CAZymes in F. oxysporum and other soilborne pathogens not only have the role of virulence factors, but also have the function of pathogen-associated molecular patterns (PAMPS), which can induce plant immune response (26–28). Most of the CAZymes identified in FCN23 belong to the GHs family with glycoside hydrolase domain, which indicates that there may be many protein hydrolases related to pathogen infection or plant immune induction in the GHs.

To further analyze the genes associated with pathogenicity, the transcriptome of FCN23 during infection of lotuses was analyzed and 7,013 DEGs were identified. GO and KEGG enrichment analysis of the upregulated genes suggests prevalence of genes associated with biosynthesis of amino acids, cofactors, and carbon metabolism during infection. Carbon metabolism plays an important role in plant cell wall degradation, and amino acid metabolism is essential for pathogen growth. Studies have shown that several genes involved in the biosynthesis of amino acids, cofactors and carbon metabolism are necessary for complete pathogenicity. During the interaction between pathogenic fungi and host plants, effectors proteins secreted by fungi are an important weapon for successful infection of plants (29, 30). We predicted 296 hypothetical effector proteins in this study. F23a002499 encoded 131 amino acids, belongs to the fungal type RNase, and was found to trigger a significant hypersensitive response by transient expression in N. benthamiana. Bioinformatic analysis revealed that F23a002499 contains four cysteine residues and typical signal peptide sequences at the N-terminal. Secreted ribonucleases play a variety of roles in many different aspects of host pathogen interactions in plant systems. Recent studies have shown that plant pathogens such as Zymoseptoria tritici (31), *Blumeria graminis* f. sp. *hordei* (32, 33), and F. graminearum (34, 35) secrete several RNase-like effectors, which contribute to the pathogenic process of pathogens.

*F. commune* is an important soilborne plant pathogen, but there is limited research on how it causes aquatic plant Fusarium wilt. In this study, the high-quality assembled genome of *F. commune* isolated from aquatic lotuses in China was obtained using Illumina and PacBio sequencing platforms. In addition, we analyzed DEGs associated with pathogenesis by transcriptome sequencing. The genome and transcriptome sequences of FCN23 will provide important genomic information and insights into how pathogens interfere with host immunity for the success of infection. Further functional analysis of pathogenic candidate genes will improve our understanding of the interaction between *F. commune* and aquatic plants.

## MATERIALS AND METHODS

### Fungal strain and genome sequencing.

The strain of FCN23 was isolated from diseased rhizomes of lotus plants in Jiangxi province of China (26^°^50′14”N, 116^°^19′32”E), and purified by single-spore subcultures on potato dextrose agar (PDA) for morphological identification. For molecular identification, the intergenic spacer region (IGS) and part of the translation elongation factor 1-alpha (*EF-1α*) were amplified and sequenced with primer pairs CNS1/CNL12 (36) and EF1/EF2-21 (37). The resulting sequences were deposited in GenBank (accession numbers ON642544 and ON642545). A phylogenetic analysis was performed using MEGA7 software with 1,000 bootstrap replicates. The FCN23 was further identified with specific primers (efFc100F and efFc385R) for *F. commune* and specific primers (FOF1 and FOR1) for F. oxysporum (38, 39). In the pathogenicity test, healthy lotus plants (cv. Taikong lotus 36) at the three-leaf stage were inoculated by dipping the roots into a conidial suspension of 1 × 10^6^ conidia/mL for 3 h. The wounds of roots were inoculated with sterile distilled water as negative controls. The experiment was performed independently three times.

The strain was cultured on PDA medium at 25°C, and fresh mycelia were harvested and ground in liquid nitrogen. Total DNA of FCN23 was isolated from the mycelia using the EZNA Fungal DNA Kit (Omega Bio-Tek, USA) following the manufacturer’s instructions. The genome sequencing was performed using the Illumina NovaSeq and the PacBio Sequel II sequencing platform. For Illumina sequencing, the library was constructed with the initial amount of 1 μg DNA, and the DNA was interrupted to 300~500 bp with a Covaris M220 Focused Ultrasonicator (Covaris Inc, Woburn, MA, USA). The sequencing library was constructed according to the TruSeq DNA Sample Prep Kit (Illumina, San Diego, CA, USA) method. The Illumina NovaSeq platform was used for whole-genome sequencing. For PacBio sequencing, genomic DNA was sheared to 15–20 kb using G-tubes (Covaris Inc, USA) and converted into the proprietary SMRT Bell library format using the RS DNA Template Preparation Kit (Pacific Biosciences, Menlo Park, CA). The library was sequenced on the PacBio Sequel II platform.

### Genome assembly.

In order to make the subsequent assembly more accurate, low-quality bases and sequencing adapter sequences were trimmed and filtered from the raw Illumina reads using Trimmomatic v0.39 (40). The following operations were performed: (i) reads with a certain proportion of low quality (less than Q20) bases were removed, (ii) reads with the proportion of N bases up to 10% were removed, (iii) the adapter and the small fragments less than 75 bp in length after quality trimming were discarded. The following operations were performed in order to filter the raw data of the PacBio Sequel II platform: (i) polymerase reads with length <200 bp were filtered out, (ii) polymerase reads with quality score <0.80 were filtered out, (iii) adapter sequences were filtered out, then subreads were produced, (iv) subreads with length <200 bp were filtered out for further analysis. The clean data was then analyzed by k-mer (k-mer = 21) to estimate the genomic size, heterozygosity, and repetition rate (41).

Canu v2.1.1 (42) and MaSuRCA v3.4.2 were tested to assemble the FCN23 genome. Canu used Pacbio long-reads to build contigs, while MaSuRCA combined both Illumina reads and PacBio long-reads. Racon v1.3.3 and Pilon v1.23 were used for further error corrections.

### Non-encoded RNA and repetitive elements analysis.

RNAmmer v1.2 (43) and tRNAscan-SE v2.0 (44) were used to predict the rRNA and tRNA contained in the genome. Infernal v1.1 (45) was used to make other kinds of ncRNA predictions based on the Rfam database and to classify statistics. The repeats were identified by RepeatMasker software (https://github.com/rmhubley/RepeatMasker). Tandem repeats finder (TRF) searches for tandem repeats in DNA sequences. RepeatMasker searches for scattered repetitive sequences by comparing sequences with a known repetitive database. The series repeat sequence was simulated by using the TRF software to copy the indel frequency by percentage and the adjacency mode, and the series repeat sequence was identified using statistical standards.

### Protein-coding genes analysis.

We identified the protein coding genes in the FCN23 genome by using a combination of *de novo* prediction, homology-based prediction, and transcriptome prediction. For *de novo* prediction, gene prediction was performed with Augustus v3.2.3 using F. graminearum as the training set (46). For homology-based prediction, the protein sequences of homologous species were aligned against the matching proteins using Genewise v2.4.1 (47). In addition, RNA-seq data generated in this study were mapped to the FCN23 genome using TopHat v2.1.1, and transcriptome-based gene structures were obtained by Trinity v2.11.0. Finally, all gene evidence was integrated using EVidenceModeler (http://evidencemodeler.github.io/) (48). The gene set integrity was then evaluated with BUSCO software (49). The protein sequences of the predicted genes were compared with NCBI Non-Redundant (NR), Kyoto Encyclopedia of Genes and Genomes (KEGG), Swiss-Prot protein databases, and Gene Ontology (GO) databases by BLASTp (BLAST + 2.7.1 with E-value cut-off <1e–05.).

### Comparative genomics and PCR verification.

The protein sequences of the FCN23 genome were compared with other fungi genomes downloaded from the Ensembl Fungi and NCBI databases. OrthoVenn (https://orthovenn2.bioinfotoolkits.net/) was used to analyze the orthologs, co-orthologs, and in-paralog pairs, as well as gene clusters function annotation (50). The tools were executed with the E-value cutoff of 1e-5 for all-to-all protein similarity comparisons, and an inflation value of 1.5 for the generation of orthologous clusters using the Markov Cluster Algorithm. TBtools v1.098684 was used to analyze the gene distribution characteristics of core and lineage-specific chromosomes (51). We further verified the results of the putative chromosome assembly by PCR amplification. Twelve genes were randomly selected for PCR amplification using the FCN23 genomic DNA as template. The primer sets were listed in Table S4.

### CAZymes and secondary metabolism gene clusters.

The identification and annotation of carbohydrate active enzymes were performed on the dbCAN2 meta server (E-Value < 1e-15, coverage > 0.35) (52). Gene clusters related to secondary metabolite biosynthesis were identified using the antibiotics and secondary metabolite analysis shell (antiSMASH v6.0) online tool (https://fungismash.secondarymetabolites.org) (53). The parameter settings were maintained as the default parameter values.

### Secretome and putative effectors.

The signal peptides of FCN23 amino acid sequences were screened using SignalP 5.0 (54). The transmembrane domain of proteins with N-terminal signal peptides were analyzed by TMHMM 2.0. TargetP 2.0 was used to predict the subcellular localization of proteins, and proteins with signal peptides located in organelles were excluded (55). PredGPI (http://gpcr.biocomp.unibo.it/predgpi/) was used to predict glycosyl phosphatidyl inositol anchor signals and retain non-GPI-anchored proteins.

In order to obtain more reliable effector proteins, we further screened these secreted proteins. The EffectorP 3.0 (http://effectorp.csiro.au/) was used to distinguish effector proteins and secreted proteins in the FCN23 genome (56, 57). Fungal effectors have the characteristics of signal peptide, low molecular weight and rich cysteine residues (58, 59). Amino acid length (≤500) and the number of cysteine residues (≥3) were used as screening conditions to further predict and analyze the candidate effector proteins.

### Inoculation treatment and RNA isolation.

Cultivar ‘Taikong lotus 36’ was used as the plant host. The FCN23 strain was incubated in a 250 mL-flask with 150 mL of PDB (potato dextrose broth) on a rotary shaker at 25°C at 180 rpm for 3 d, and then the spores were harvested. The spore suspension with a concentration of 10^6^ was prepared for inoculation. The young lotus whips, about 10 cm in length, were disinfected with 75% alcohol and rinsed with sterile water three times. For inoculation treatment, the lotus whip was punctured with a needle, and a 10-μL spore suspension was added to the surface of the wound. The lotus whip with sterile water was added as a control. Each treatment was repeated three times.

For transcriptome analysis of FCN23 during infection, the samples of pure culture and inoculation for 96 h were collected. RNA isolation and reverse transcription for cDNA synthesis were carried out using TRIzol reagent (Invitrogen, USA) and the PrimeScript RT reagent kit with gDNA Eraser (TaKaRa, Japan) according to the manufactory’s direction.

### Transcriptome sequencing and analyses.

A total of 1.5 μg RNA from each sample was used as input material for RNA library preparation using the NEBNext Ultra™ RNA Library Prep Kit for Illumina (NEB, USA) following manufacturer’s instructions. The library was initially quantified using the Qubit2.0 Fluorometer and then diluted to 1.5 ng/μL. qRT-PCR was used to accurately quantify the effective concentration of the library to ensure the quality of the library. The library preparations were sequenced d by the Illumina NovaSeq 6000.

The raw reads were preprocessed by removing low-quality reads and adaptor reads using SOAPnuke (60). All the high-quality reads were aligned against the reference genome of strain FCN23 using HISAT v2.0.5 (61). The data of RNA-seq has been deposited in NCBI Short Read Archive database (BioProject number PRJNA795051).

### Gene expression and quantitative RT-PCR.

According to the position information of gene alignment on the reference genome, the number of reads covered by each gene from start to end was counted using featureCounts v1.5.0-p3 (62). Reads with a comparison quality value lower than 10, reads on noncomparison pairs, and reads in multiple regions of the genome were filtered out. Gene expression levels were estimated by the fragments per kilobase per million mapped fragments (FPKM) for each sample. The differential expression analysis was performed using the DESeq2 R package v1.20.0 (63). Genes with an adjusted padj<=0.05 and | log_2_(foldchange)|>=1 were assigned to be differentially expressed. Gene Ontology (GO) enrichment analysis of the differentially expressed genes (DEGs) was implemented by the clusterProfiler R package v3.8.1, in which gene length bias was corrected (64). GO terms with corrected *P* values less than 0.05 were considered significantly enriched by DEGs. Then we used clusterProfiler software to test the statistical enrichment of differential expression genes in KEGG pathways.

For quantitative RT-PCR, inoculated samples were collected at 0, 6, 12, 24, and 36 h postinoculation (hpi). Reactions were performed on Bio-Rad CFX96 real-time PCR detection system with the SYBR qPCR Master Mix (Transgen Biotech, China). For each gene, three biological replicates were prepared for qPCR analysis and the average threshold cycle (Ct) was calculated. The relative gene expression was calculated using the 2^-ΔΔ^Ct method. The translation elongation factor 1-α (*EF-1α*) gene of FCN23 was chosen as the endogenous reference gene. The F23a002499 gene was amplified with 499F (5'ACGACGACACTGCTGGAA3') and 499R (5'TAAACACCGCCGCTCTTG3'), while the *EF-1α* gene was amplified with EF1nF (5'TCAGGGTGCCGCTTCT3') and EF1nR (5'CTTGACGATGGCGGAGT3').

### Transient expression analysis of candidate effectors in Nicotiana benthamiana.

The candidate effector genes (without signal peptide regions) in the FCN23 genome were amplified from the cDNA library using KOD FX DNA polymerase (Toyobo, Osaka, Japan). The primer sets were listed in Table S12. The expression vector pBinGFP was linearized by digestion with KpnI and XbaI. Purified PCR product was cloned into pBinGFP by using ClonExpress MultiS One-Step Cloning Kit (Vazyme, China). Then these constructs were transformed into the Agrobacterium tumefaciens strain GV3101 using the freeze-thaw method.

The verified individual colonies of A. tumefaciens were cultured in LB medium containing rifampicin (10 μg/mL) and kanamycin (50 μg/mL) for 24 h. Then bacterial cells were centrifuged at 5000 rpm for 5 min, washed three times with 10 mM MgCl_2_, and resuspended in MMA buffer (10 mM MgCl_2_, 10 mM MES, 150 μM acetosyringone, pH 5.6). The OD_600_ was adjusted to 0.5 and left in a dark at room temperature for 3 h. Leaves of 4-week-old N. benthamiana plants were inoculated by needleless syringes as previously described (65). The pBinGFP was used as the negative control, while the addition of the proapoptotic protein BAX was used as the positive control that induced cell death. Cell death symptoms were evaluated and photographed at 3 days after infiltration. The experiments were repeated at least three times.

### Data Availability.

The entire genome of Fusarium *commune* FCN23 has been deposited at DDBJ/ENA/GenBank under the accession number JAJTCY000000000. The data of RNA-seq has been deposited in NCBI Short Read Archive database (BioProject number: PRJNA795051).
