# Supplementary material for: Genome and Transcriptome Sequencing Analysis of Fusarium commune Provides Insights into the Pathogenic Mechanisms of the Lotus Rhizome Rot
Source: Microbiol Spectr. 2022 Jul 5;10(4):e00175-22. doi: 10.1128/spectrum.00175-22 (PMC9431280; doi:10.1128/spectrum.00175-22)
Supplement: Supplemental file 2 — Supplemental material. Download spectrum.00175-22-s0002.pdf, PDF file, 0.9 MB [file spectrum.00175-22-s0002.pdf]

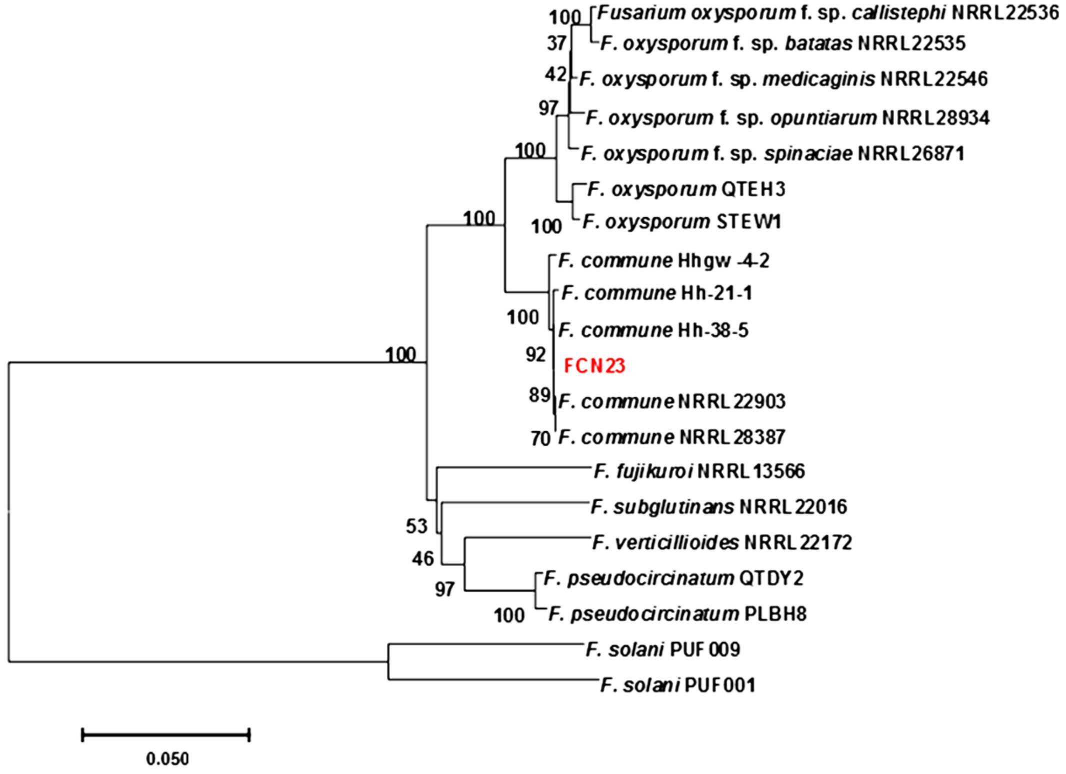

Figure S1. Neighbor-Joining tree generated from combined ribosomal intergenic spacer (IGS) and translation elongation factor 1-alpha (*EF-1a*) sequences.

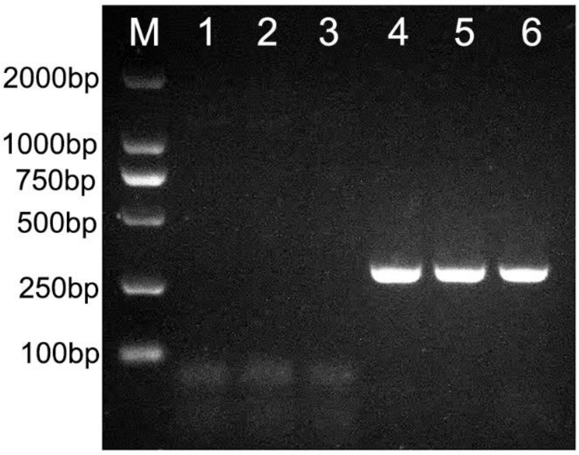

Figure S2. The FCN23 isolate from lotus root in China were vertified by species-specific diagnostic primers. M: DNA marker 2000. 1-3: PCR products of FCN23 gDNA using FOF1/FOR1 primers developed by Mishra et al. for detection of *F. oxysporum*. 4-6: PCR products of FCN23 gDNA using efFc100F/efFc385R primers developed by Stewart et al. for detection of *F. commune*.

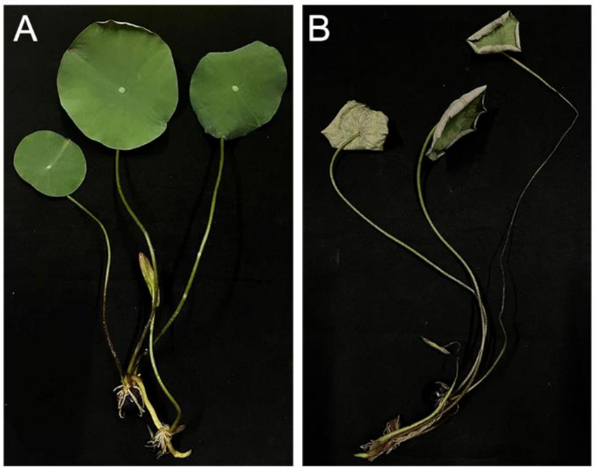

Figure S3. Pathogenicity tests of FCN23 on lotus root. (A) The wounds of roots were inoculated with sterile distilled water as negative controls. (B) The typical wilt symptoms appeared after five days of inoculation at 25°C.

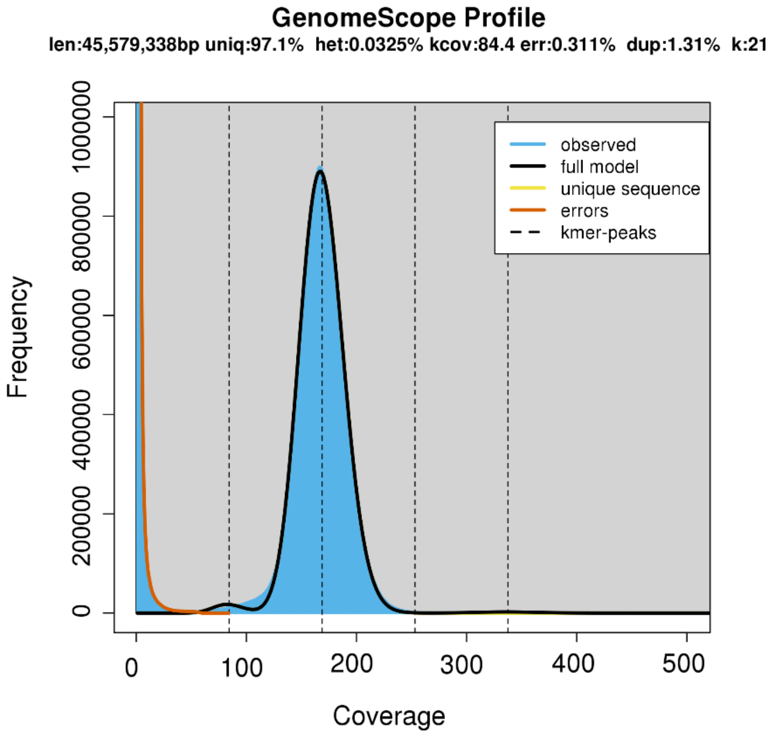

Figure S4. K-mer (k=21) analysis of FCN23 genome using Jellyfish software.

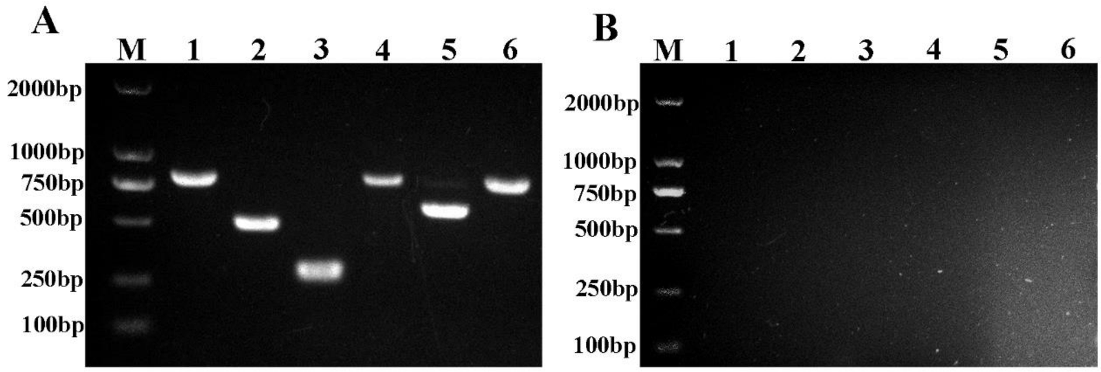

Figure S5. PCR validation of FCN23 genome assembly results. (A) Six unique genes of FCN23 were amplified by PCR using FCN23 genomic DNA as template. M, DNA marker 2000. Lane 1 and 2: F23a004339.1 and F23a013161.1 genes located on putative chromosome 6 of FCN23 genome; Lane 3: F23a013131.1 gene located on putative chromosome 14 of FCN23 genome; Lane 4-6, F23a014275.2, F23a012984.1 and F23a014237.2 genes located on putative chromosome 15 of FCN23 genome. (B) Six genes located on chromosome 3 of Fol4287 were amplified by PCR using FCN23 genomic DNA as template. Lane 1-6, KNB20228, KNB20229, KNB20239, KNB20257, KNB20258, KNB20265 genes of Fol4287 located on chromosome 3 of Fol4287.

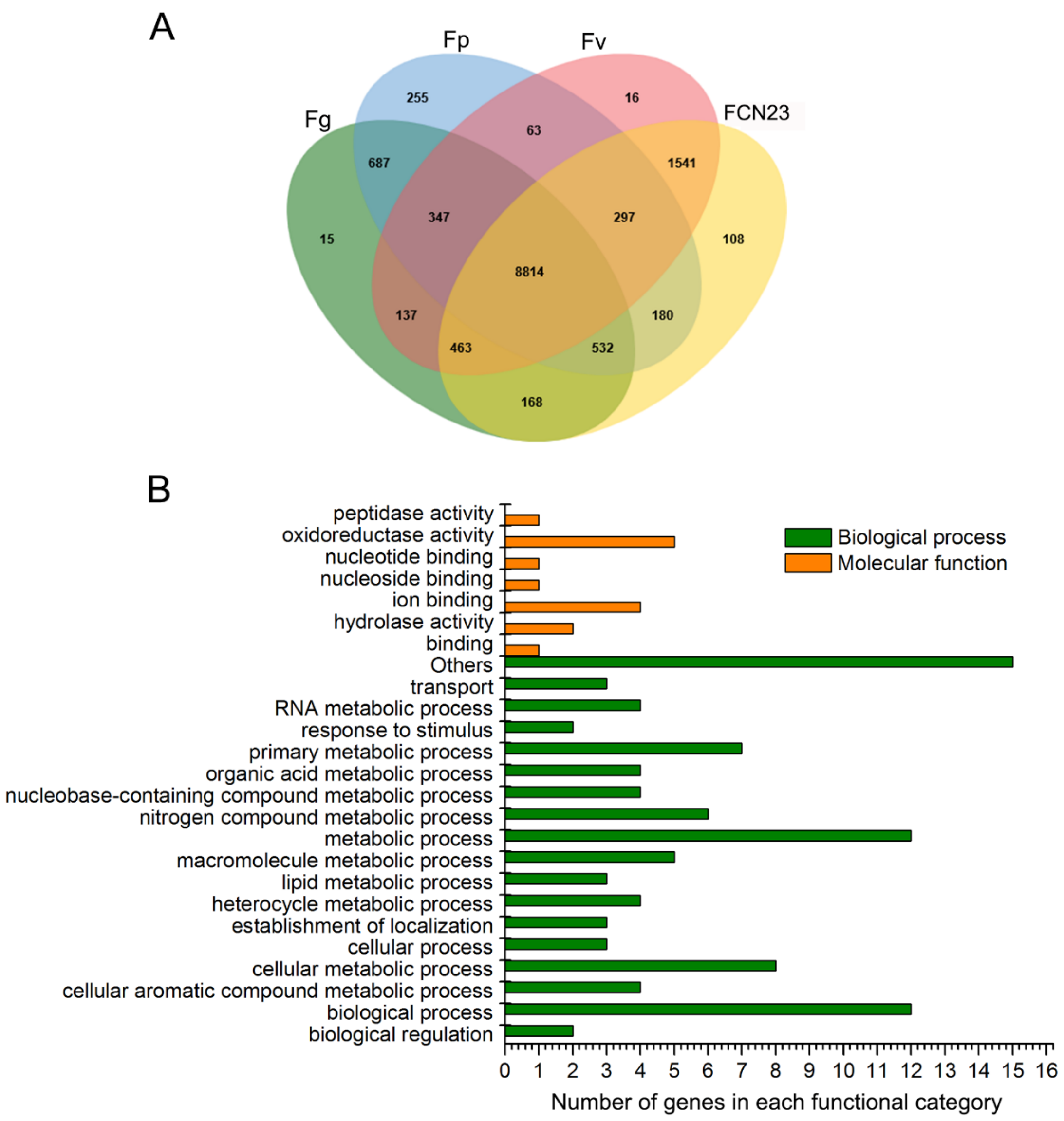

Figure S6. Comparison of orthologous between FCN23 and three other *Fusarium* species. (A) Venn diagram of gene clusters, FCN, *F. oxysporum* f. sp. *nelumbicola*; Fg, *F. graminearum*; Fp, *F. poae*; Fv, *F. verticillioides*, *F. verticillioides*. (B) Go enrichment analysis of annotated genes only exists in FCN23 genome but not in three other *Fusarium* species.

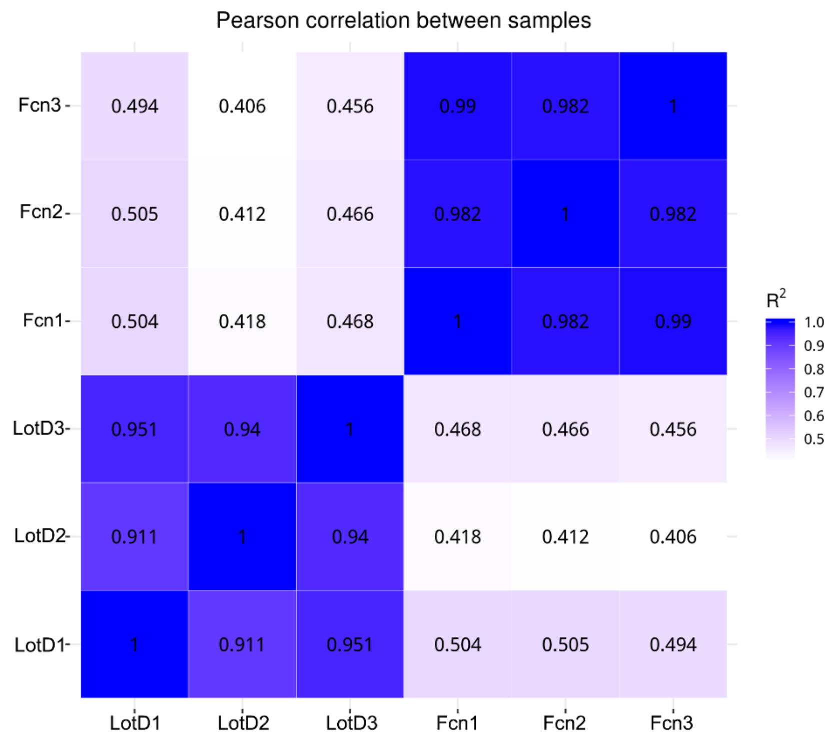

Figure S7. Heat map of the correlation between three biological replicates of different treatments.

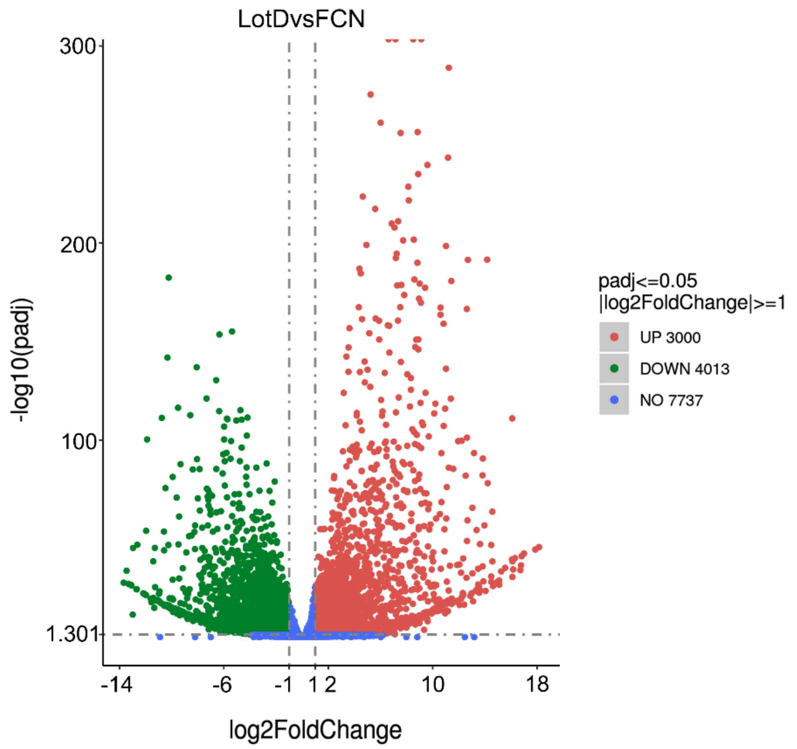

Figure S8. Volcano of differentially expressed genes at 96 h post inoculation to the lotus 'Taikong lotus 36'.
